# A Two-Stage Mohs Micrographic Surgery Technique to Avoid Complex Reconstruction of Large Skin Lesions

**DOI:** 10.3390/life16061005

**Published:** 2026-06-15

**Authors:** Ariel Berl, Ofir Shir-az, Biader Samih Bilal, Din Mann, Avshalom Shalom

**Affiliations:** 1 Department of Plastic Surgery, Meir Medical Center, Kfar Saba 4428164, Israel; arielberl23@gmail.com (A.B.); ofir.shiraz@gmail.com (O.S.-a.); biader.bilal@gmail.com (B.S.B.); dinmannlit@gmail.com (D.M.); 2 Gray School of Medicine, Faculty of Medical and Health Sciences, Tel Aviv University, Tel Aviv 6997801, Israel

**Keywords:** facial esthetics, Mohs micrographic surgery, skin cancer, surgical technique, serial excisions

## Abstract

Mohs Micrographic Surgery (MMS) is considered the most conservative and preserving procedure for removing cutaneous tumors. The major disadvantage of MMS is that tumor involvement in tissue may be underestimated. This may lead to large excisions necessitating complex reconstruction with profound effects on cosmetic results. Some patients refuse complex reconstruction and demand simple closure of post-MMS skin defects. This retrospective cohort study describes our technique of serial Mohs excisions of large non-melanoma skin cancers for patients refusing flaps or skin graft reconstructions. A total of 51 patients who underwent MMS according to the described technique February 2020–May 2021 were included. The mean age was 76.5 (range 63–94) years and 55% were male. More than half of the lesions were on the nose. Mean lesion sizes were 14.25–55 mm depending on location. Most cases required two surgeries and only one needed a third surgery. Postsurgical defects were repaired using primary closure in 90% of cases. Mean follow-up was 31 months (range 6–48) with no evidence of local recurrence. In conclusion, this approach of serial excisions with MMS can be performed safely and achieve better cosmetic outcomes for patients presenting with large skin tumors of the face or other functionally important areas.

## 1. Introduction

Mohs Micrographic Surgery (MMS) was first described by Frederic Mohs in 1938 [1] and is considered the most conservative and tissue-preserving procedure for the complete removal of cutaneous tumors. The use of MMS has increased steadily and is considered by many to be the gold standard for treating non-melanoma skin cancers (NMSC) in esthetic and functionally sensitive areas [2]. This technique has undergone many refinements throughout the years and has evolved into the procedure we know today [1,3,4,5].

Modern MMS has many advantages, including immediate and complete excision of tumor with clear margins, while preserving the maximum amount of uninvolved tissue. It is cost-effective and safe, with minimal complications [2,6,7,8,9]. The major disadvantage of MMS is that the degree of tissue involvement may be underestimated due to the inherent nature of skin tumors to extend beyond visible borders [2]. When operating on sensitive areas of the face, any extension of the excision may require complex reconstruction and can have a profound effect on cosmetic results. Some patients refuse to undergo complex reconstruction and demand a simple closure of post-MMS skin defects.

Staged excision of NMSC has been described previously and found to be oncologically safe in cases that required preservation of healthy tissue [10,11,12,13].

This study describes our technique for serial Mohs excisions of large skin lesions in patients refusing complex reconstruction, including flaps or skin grafts.

## 2. Materials and Methods

Patients who presented for removal of NMSC skin lesions from February 2019 to May 2021 were eligible for inclusion in this retrospective cohort study. Data were abstracted from comprehensive electronic medical records. All surgeries were conducted in one operating center by the senior author.

The patients were thoroughly examined for suspected skin lesions, clinically and dermatoscopically. They were told that large lesions in esthetic or functionally sensitive regions might require complex reconstruction. All those who refused complex reconstruction were eligible to undergo the described technique and to be included in this study.

All the candidates received a detailed explanation of the procedure and of the need for close follow-up and additional surgeries. Lesions suspected as basal cell carcinoma (BCC) were eligible. It is imperative to thoroughly explain the procedure to the patients and assure they understand the need for future surgeries.

### 2.1. Data Analysis

Descriptive and univariate statistical analyses were performed using SPSS-21 (IBM Corp., Armonk, NY, USA). A *p*-value < 0.05 was considered significant. Data were collected on an Excel spreadsheet (Microsoft Office, Redmond, WA, USA) and were double checked and corrected for errors to ensure data quality.

### 2.2. Operative Technique

The technique presented here involves serial excisions of skin tumors of the facial or functionally important regions, using MMS. The initial excision is carried out in the same manner as the traditional MMS, beginning with the lateral border and cutting towards the midline of the lesion. As opposed to the traditional approach, we excise as much tissue as possible, while ensuring that a primary closure is possible. A simple pinch test can provide a good estimation of the amount of tissue that can be excised in each stage. The more experienced surgeon can rely on experience and simple examination of skin elasticity, whilst if unsure, approximating sutures can be used. The guiding principle is removal of the largest amount of tissue possible and primary closure without causing a visible deformation. The blade is replaced and the cut specimen is then excised laterally to medially.

The tissue is prepared for analysis and margins are examined under a microscope. The lateral border is examined thoroughly, and the margin is re-excised until at least one side is clear of tumor cells. Ensuring that at least one margin is clear will assist in future stages. It is important to only remove tissue to the extent that will allow a primary closure of the wound without distorting surrounding tissue. The skin defect is then closed with monofilament, non-absorbable sutures. The patients are re-examined at the office on postoperative day (POD) 7 and assessed for complications and tissue healing. The sutures are removed during this visit.

**Figure 1:**
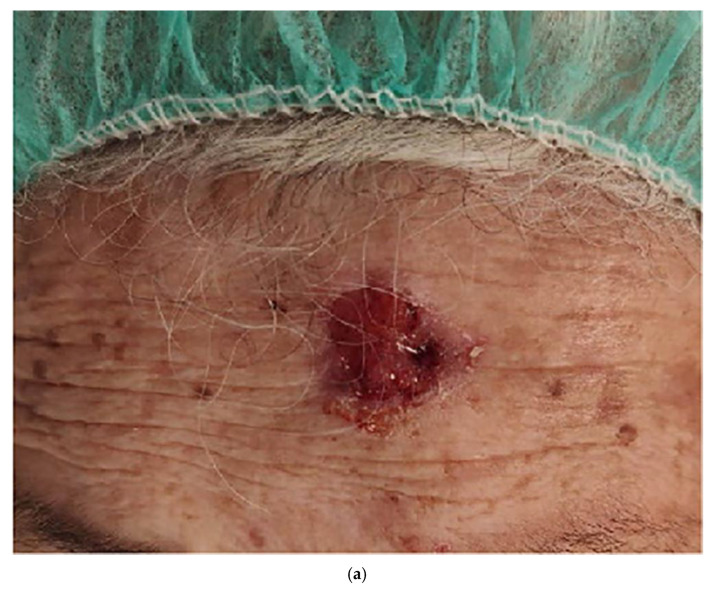

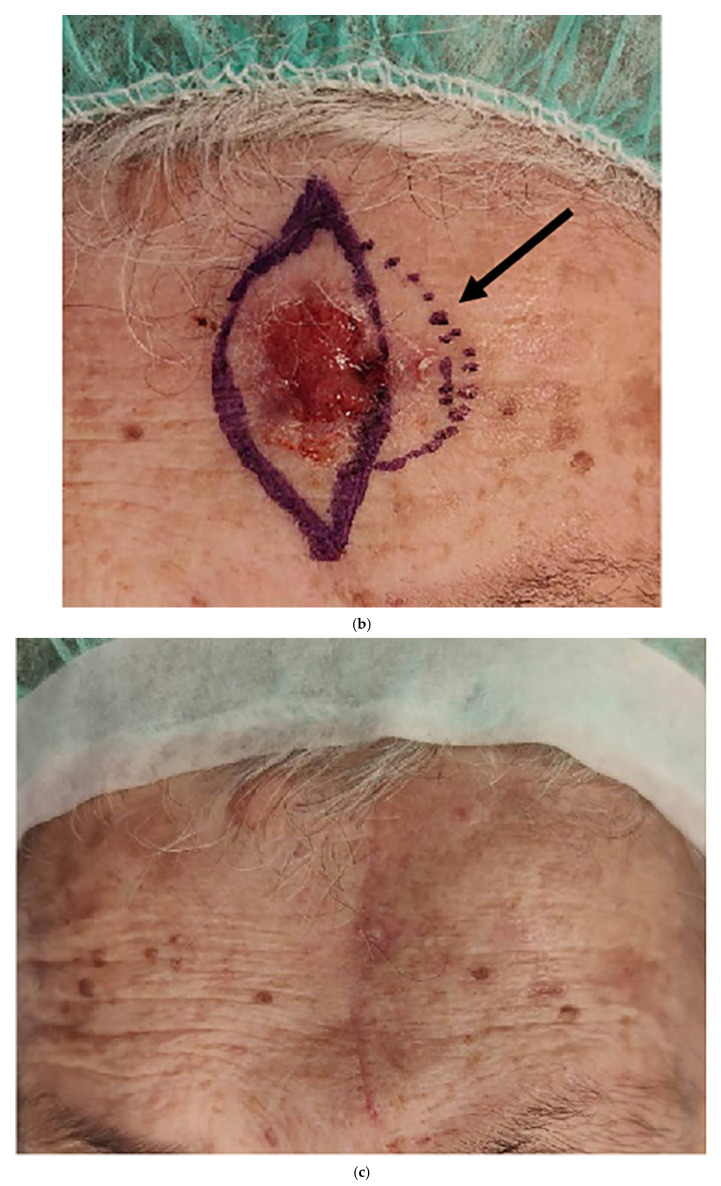

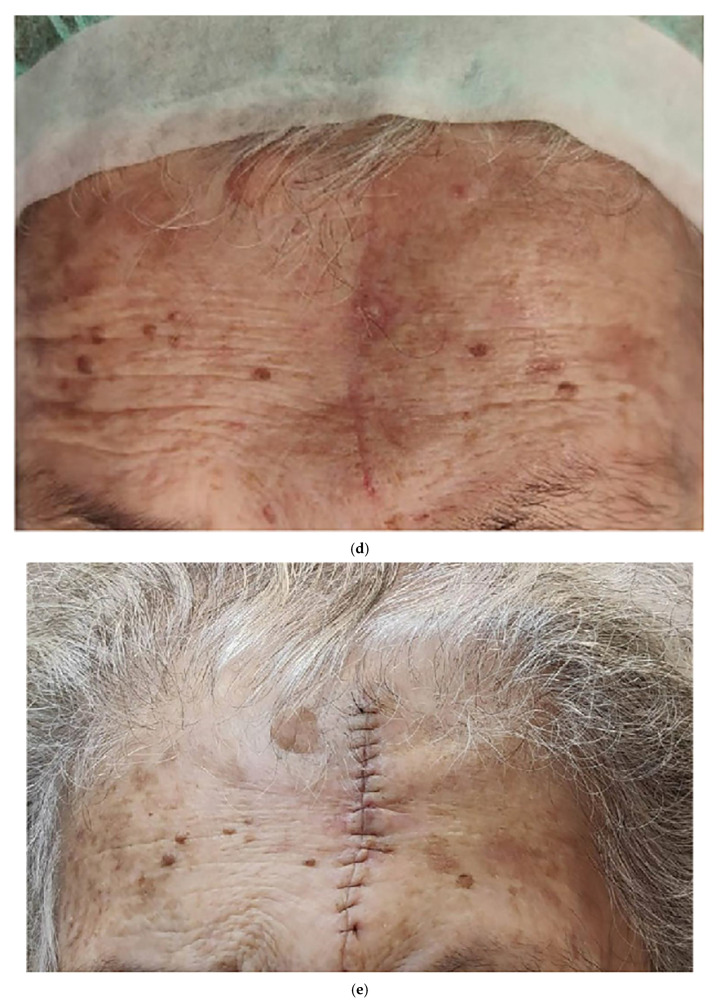
An 87-year-old patient with a large (30 mm), ulcerated lesion on her forehead and a small co-lesion on the left (black arrow). (**a**) Presurgery; (**b**) the larger lesion marked for excision at the first stage; (**c**) 8 weeks after the first stage, the small co-lesion was still present (arrow); (**d**) the second lesion is marked for excision; (**e**) the patient on POD 7 following the second stage.

**Figure 2:**
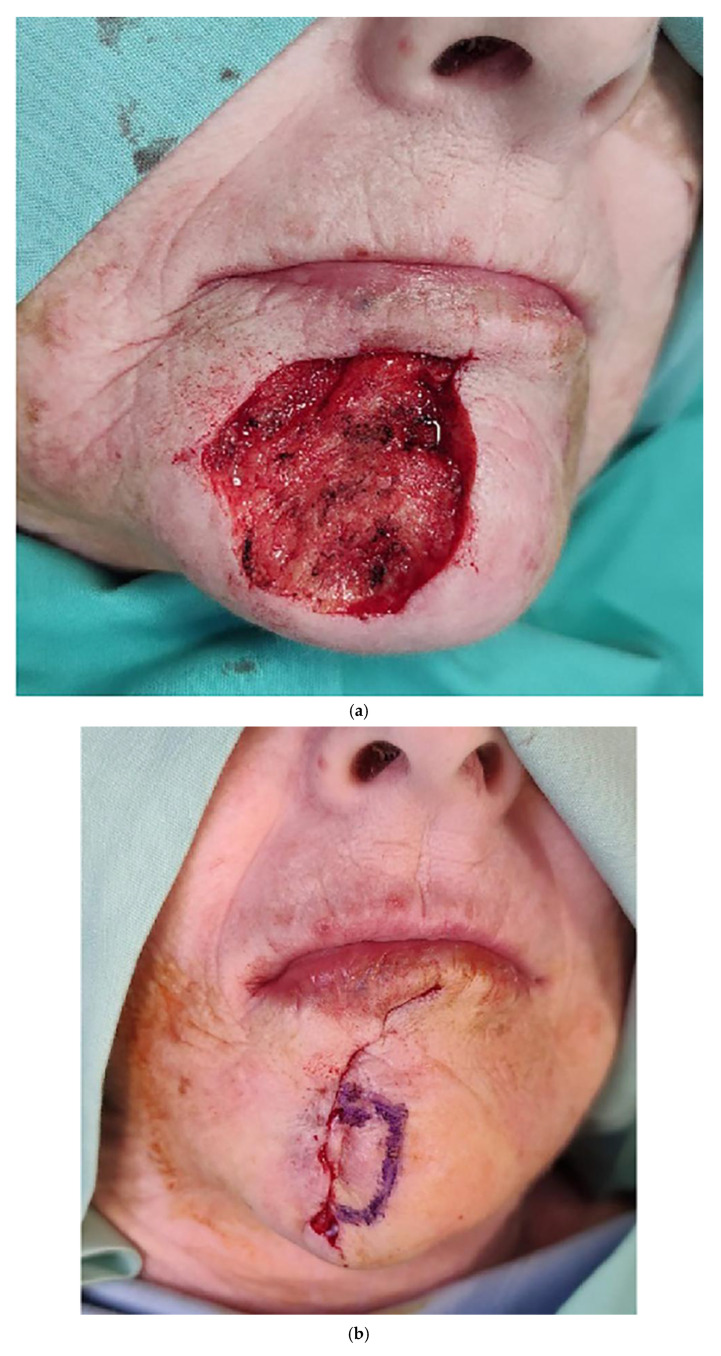

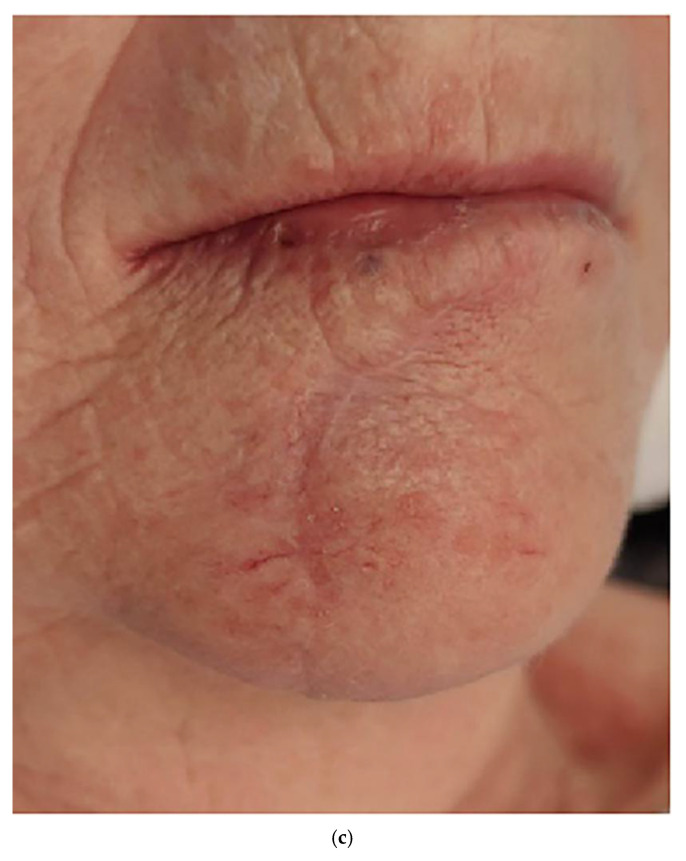

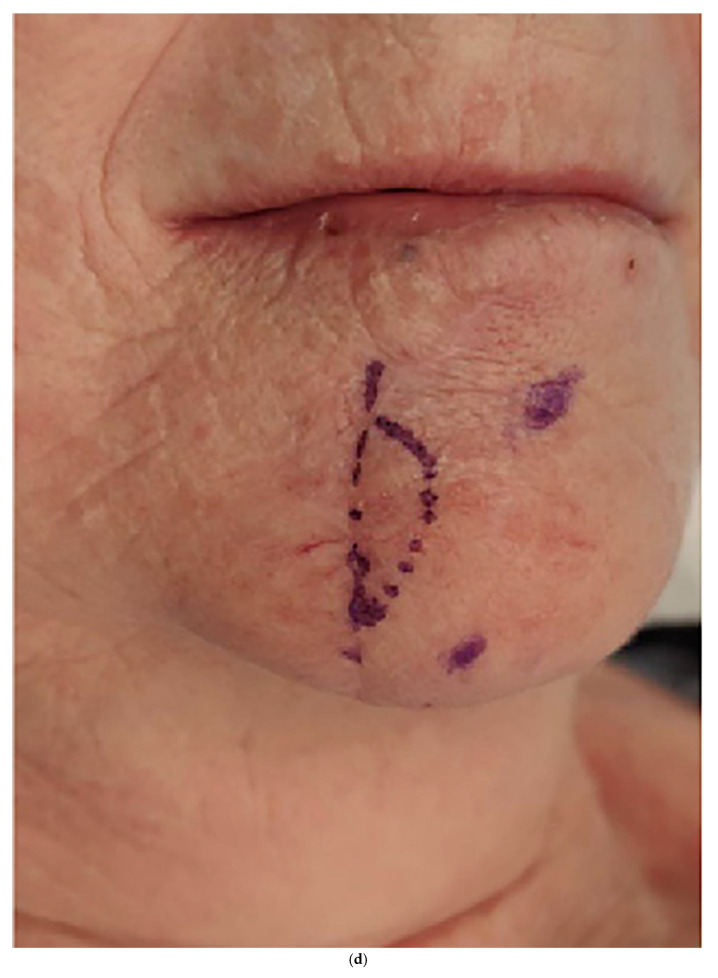

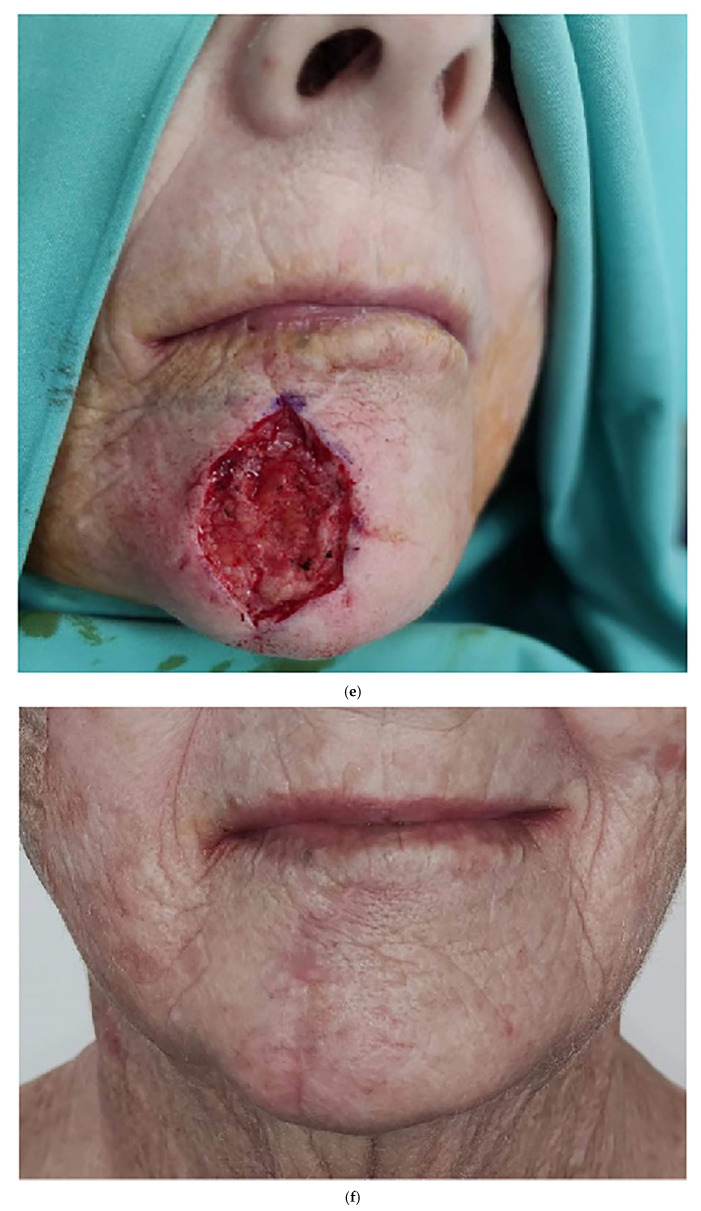
An 82-year-old patient who presented with a lesion on her chin. (**a**) The surgical defect after 3 MMS stages; (**b**) the primary closure of the defect—the medial border with involved margin is marked; (**c**) 8 weeks after the first stage; (**d**) the medial border that was involved is remarked; (**e**) the surgical defect after completion of the second stage; (**f**) the patient at the 6-month follow-up visit.

**Figure 3:**
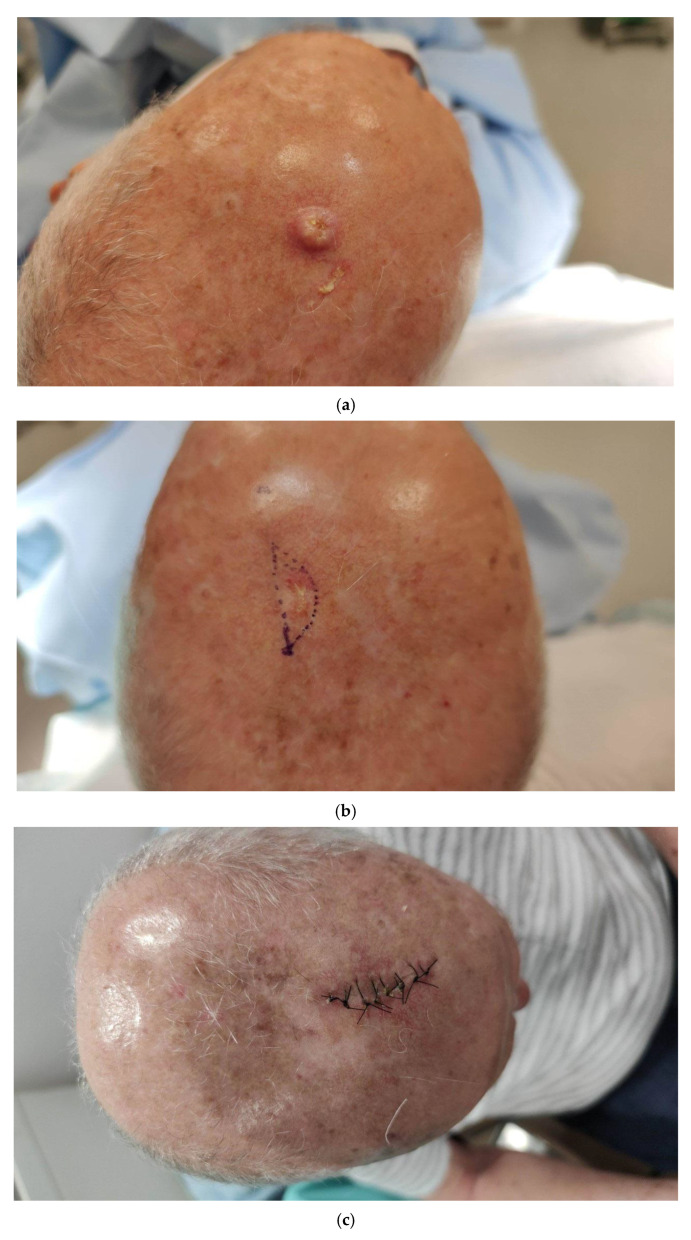
A 72-year-old patient who presented with a lesion on his scalp. (**a**) Presurgery; (**b**) marking for the second-stage excision; (**c**) the patient on POD 10, following the second stage.

The second stage of the procedure is carried out after a minimum of six weeks in the same manner as the first, with the exception that both the lateral and the initial medial borders are examined and re-excised until free of tumor cells. In the case of extremely large lesions, further serial excisions can be considered after discussing the process with the patient (Figure 1a–e, Figure 2a–f and Figure 3a–c).

## 3. Results

A total of 51 patients who underwent MMS during the study period, according to the technique described, were included. The mean age was 76.5 years (range 63–94) and 55% were male. One patient was lost to follow-up and two patients died from unrelated causes after the initial surgery.

Lesions were located on the nose (29, 56%), forehead (7, 14%), scalp (5, 10%), cheek (4, 8%), neck (3, 6%), chin (2, 4%), and leg (1, 2%). No patient had more than one lesion excised in a single visit. The mean initial size of lesions in mm according to location were nose 14.3, forehead 23.9, scalp 22.0, cheek 23.3, neck 19.5, chin 55.0, and leg 20.0.

Fifty (98%) patients required two MMSs and only one patient required a third surgery. The mean number of stages in the first, second and third visits were 1.1, 1.2 and 1.0, respectively.

Postsurgical defects were closed by primary closure in 45 (90%) cases and six (11.7%) required a simple local flap (hatchet, transposition or nasolabial). Post-operative follow-up of patients included a visit on POD 7 for suture removal, and at 1 month and 1 year after surgery. The average follow-up period was 13 months. No major complications, such as dehiscence or infections, were seen during the follow-up period.

Histopathological analysis of the lesions included 42 (82.4%) BCCs and nine (17.6%) Bowen’s. BCC subtypes included 21 morphea, 16 superficial, seven nodular, six pigmented and one metatypical. A total of 17 BCCs had an ulcerated appearance (see Table 1 for further details). None of the patients experienced local recurrence.

All the patients were seen in the clinic for suture removal on POD 7–10. The mean follow-up period was 31 months (range 6–48) with 70% of patients completing at least 6 months of follow-up. No clinical or dermoscopic local recurrence was seen in any of those who continued to follow-up.

## 4. Discussion

Mohs Micrographic Surgery (MMS) is the standard of care for removal of NMSC from esthetically and functionally important areas. This technique has several advantages compared to standard serial excisions [2,6,7]. One disadvantage of MMS, especially in medium-to-large lesions on the face, is that the extent of involved tissue is unknown [14]. When delicate, esthetic regions of the face are involved, the removal of even small amounts of tissue may necessitate complex reconstructions, leaving visible and sometimes disfiguring scars.

The technique presented here is the result of patients refusing to undergo complex reconstruction, and demanding simple closure of a post-MMS skin defect instead. The technique described here combines the major advantages of the traditional MMS, while controlling the final amount of tissue removed by using serial excisions.

Our experience with 51 patients showed that most cases (45, 90%) were closed using primary closure and only six cases necessitated a simple local flap (two hatchet, three transposition and one nasolabial). Closure of a wound in a simple, primary manner circumvents the need for complex reconstructions including skin grafts and flaps, which are time-consuming, costly and can be unaesthetic. Staged excision of NMSC of the face has been proven to be safe from an oncological perspective [12,13]. Therefore, it can be offered to this subset of patients following a thorough explanation and close follow-up.

The method presented here can eliminate the need for an intricate reconstruction with visible results. The use of staged excision takes advantage of the inherent nature of skin and the stress relaxation phenomenon, as the skin stretches over time due to tension and various forces acting on the skin before it relaxes and settles into place [15,16]. Accordingly, this will aid the surgeon in the subsequent excision, when removing tissue and closing with minimal tension. In most cases, BCC is slow-growing and distant spread is rare [17,18]. Staged excision is an acceptable approach for the removal of BCC and is considered safe from an oncologic perspective [12,17,19,20].

It must be emphasized that this technique is suitable for patients with BCC and Bowen’s Disease, and not for invasive SCC or other suspected aggressive tumors. As presented above, there is a minimum six-week period between stages of excision and some might argue that the tumor will progress during this period. In our opinion and relying on published data, this is a safe approach and is also close to the time patients wait to receive tissue biopsy results and then schedule and undergo surgery.

This study had a few limitations. It was retrospective and all the surgeries were conducted by a single surgeon, which may result in bias and limit its generalizability to other clinical settings.

Although previous studies have described staged and serial excision of NMSC, this paper provides a complete description of the technique along with our experience showing that it is safe, efficient, cost-effective, and reproducible.

In our experience, serial excisions for treating large NMSC while avoiding the need for complex reconstruction are suitable for a subset of patients, whether they request it or the surgeon prefers this approach for a particular case. This method can also be used when incisional biopsies are required in esthetically or functionally important areas. Removing the maximum amount of tumor possible during the biopsy while still allowing for primary closure can assist in the subsequent stages of completing the excision. The cohort of patients described here represents about 2% of the patients treated in this specific MMS clinic. This strengthens our belief that this modality is suitable only for a specific subset of patients.

When approaching more esthetically problematic areas such as the ear, we did not use the described technique, as this area is seldom closed by primary closure and may require complex reconstructions such as skin grafts and local flaps. As for the nose, the presented technique was implemented with excellent results.

In select cases and locations, defects can be left to heal by secondary intention. Although this is a reasonable modality in some cases, in the described technique the tumor is not completely excised in the first surgery and at least one additional surgery will be required. Leaving the wound to heal secondarily can take a long time and although it may achieve reasonable esthetic results, local tissue scarring that prevents excision and primary closure in the second stage may occur. Furthermore, healing can be prolonged in some patients, delaying the completion of tumor resection. Esthetic results can also be inferior to primary closure, making the described technique a useful and attractive modality.

As described above, it should be emphasized that there is no specific size limitation and a simple pinch test is used to guide the extent of excision at each stage. There is a learning curve in gaining experience with this technique; however, the less experienced surgeon can rely on examining skin elasticity and the use of approximating sutures. The guiding principle is to aim for removal of the largest amount of tissue possible and primary closure without causing a visible deformation.

To prevent confusion and to facilitate learning and improvement from cases, we recommend photographic documentation, with a scale, of all stages. This includes but is not limited to the lesion upon presentation, excision markings, and final scar length after each stage. The advent of high-quality cameras on phones and digital cameras linked to patient files can facilitate timely and efficient documentation for future reference.

When using this approach, we found that the cosmetic results were better, with minimal scaring, and complex reconstruction was not needed. Even though patients needed to return for a second procedure, the combined approach resulted in a smaller scar with better cosmetic results and high patient satisfaction. In our cohort with a mean follow-up of over 30 months, no clinical or dermoscopic local recurrences were noted, further emphasizing the efficacy and safety of the described technique.

We believe that among patients presenting with large skin tumors of the face or other functionally important areas, who refuse to undergo complex reconstruction following MMS, our combined approach of serial excisions, which is a fast, safe and inexpensive expansion procedure, combined with MMS can be performed safely and achieve better cosmetic outcomes.

## Figures and Tables

**Table 1:** Lesion locations and subtypes.

Variable	Tumor Location, n (%)						
	Nose 29 (56)	Neck 3 (6)	Scalp 5 (10)	Forehead 7 (14)	Leg 1 (2)	Cheek 4 (8)	Chin 2 (4)
Mean size, mm	14.25	19.5	22	23.85	20	23.33	55
Range, mm	9–25	17–22	20–30	20–30	-	20–25	54–56
Type of reconstruction							
Primary	27 (93)	3 (100)	4 (80)	6 (86)	1 (100)	3 (75)	1 (50)
Local flap	2 (7)	-	1 (20)	1 (14)	-	1 (25)	1 (50)
BCC subtype							
Morphea	16	-	2	1	-	1	1
Superficial	7	3	1	2	1	1	1
Metatypical	-	-	1	-	-	-	-
Nodular	2	-	1	2	-	2	-
Pigmented	4	-	-	2	-	-	-
Ulceration present	6	-	5	6	-	-	

BCC, basal cell carcinoma.

## Data Availability

Data will be made available upon reasonable request to the corresponding author.

## Abbreviations

The following abbreviations are used in this manuscript:

MMS	Mohs Micrographic Surgery
NMSC	Non-melanoma skin cancers
BCC	Basal cell carcinoma
POD	Postoperative day
SCC	Squamous cell carcinoma
